# Characterization of Perineuronal Nets in the Paraventricular Nucleus of the Hypothalamus and their Alteration in Neurogenic Hypertension

**DOI:** 10.1007/s10571-025-01628-z

**Published:** 2025-11-17

**Authors:** Ismary Blanco, Sichu Chen, Erin Yeo, Samantha Reasonover, Monica M. Santisteban

**Affiliations:** 1 Vanderbilt University Medical Center, 2220 Pierce Ave, RRB-536, Nashville, TN 37232 USA; 2 Vanderbilt University, 2201 W End Ave, Nashville, TN 37235 USA

**Keywords:** Hypertension, Perineuronal nets, Paraventricular nucleus of the hypothalamus

## Abstract

Perineuronal nets (PNNs) are key regulators of neuronal excitability, yet whether they are altered during neurogenic hypertension is unknown. Here, we mapped the developmental trajectory of PNNs in the paraventricular nucleus of the hypothalamus (PVN), a crucial nucleus involved in blood pressure (BP) regulation, and examined their modulation in neurogenic hypertension. We show that PNNs in the PVN follow a developmental pattern similar to other brain regions. The most prevalent neuron subtype enwrapped by PNNs was neuronal nitric oxide synthase (nNOS)-expressing neurons in both sexes, and sex differences were observed only in oxytocin (OXT)-enwrapped neurons. In the DOCA-salt mouse model of neurogenic hypertension, males, but not females, exhibit an increased number and area of PNNs in the PVN with increased excitatory/inhibitory (E/I) ratio. Given that PNNs modulate neuronal activity, our findings may implicate recruitment of previously “silent” neurons as potential contributors of PVN hyperactivity in hypertension. These results demonstrate that PNN remodeling is associated with neurogenic hypertension in male mice.

## Introduction

Hypertension affects over one billion people worldwide and more than 45% of U.S. adults (Flack et al. 2024; Kario et al. 2024). Resistant hypertension, commonly neurogenic in origin, affects up to 40% of patients (Flack et al. 2024). The latter, is driven by neurohumoral dysregulation and heightened sympathetic output (Flack et al. 2024; Lamptey et al. 2023; Zheng et al. 2022), largely orchestrated by the paraventricular nucleus of the hypothalamus (PVN) (Basting et al. 2018; Guyenet 2006). Indeed, PVN overactivation is a hallmark of neurogenic hypertension (Goncharuk et al. 2002; Guyenet 2006; Xi et al. 2024), reflecting altered excitatory/inhibitory (E/I) balance. Several studies report increased glutamatergic transmission (D.-P. Li et al. 2008; D.-P. Li and Pan 2006; Ye et al. 2012) and reduced inhibition (Y.-F. Li et al. 2001). However, the cellular and molecular mechanisms underlying PVN overactivation remain incompletely understood.

A novel mechanism that may contribute to PVN hyperactivity is alterations in perineuronal nets (PNNs), extracellular matrix structures that regulate neuronal firing (Balmer 2016; Carulli and Verhaagen 2021; Frischknecht et al. 2009; Tewari et al. 2018). PNNs are distributed throughout the central nervous system (Lupori et al. 2023), including the hypothalamus (Horii-Hayashi et al. 2017), where they typically enwrap neuronal cell bodies, proximal dendrites, axon initial segments, and synaptic terminals (Balmer 2016; Cabungcal et al. 2013; Carceller et al. 2020; Carstens et al. 2016; Carulli and Verhaagen 2021; Celio 1993; Favuzzi et al. 2017; Fawcett et al. 2019; Frischknecht et al. 2009; Tewari et al. 2018). PNNs are composed primarily of chondroitin sulfate proteoglycans (CSPGs) such as aggrecan, brevican, neurocan, and versican, cross-linked together and anchored to the cell surface (Carulli and Verhaagen 2021; Deepa et al. 2006; Fawcett et al. 2022; Galtrey et al. 2008). Of relevance to neurogenic hypertension, an increase in PNN components and PNN-enwrapped neurons is dependent on increased neuronal activity (Carulli et al. 2007; Dityatev et al. 2007). PNNs also exhibit diurnal modulation (Harkness et al. 2021) paralleling blood pressure (BP) regulation. Additionally, they are dynamically remodeled by activity-dependent processes such as learning and memory (Fawcett et al. 2022; Tewari et al. 2022) as well as by inflammation (Dong et al. 2023), which plays a pivotal role in the pathophysiology of hypertension. Based on this, we investigated which PVN neuronal cell types are enwrapped by PNNs and whether these structures are modulated in a mouse model of neurogenic hypertension.

## Results

### Perineuronal Nets are Developmentally Regulated in the PVN

**Fig. 1:**
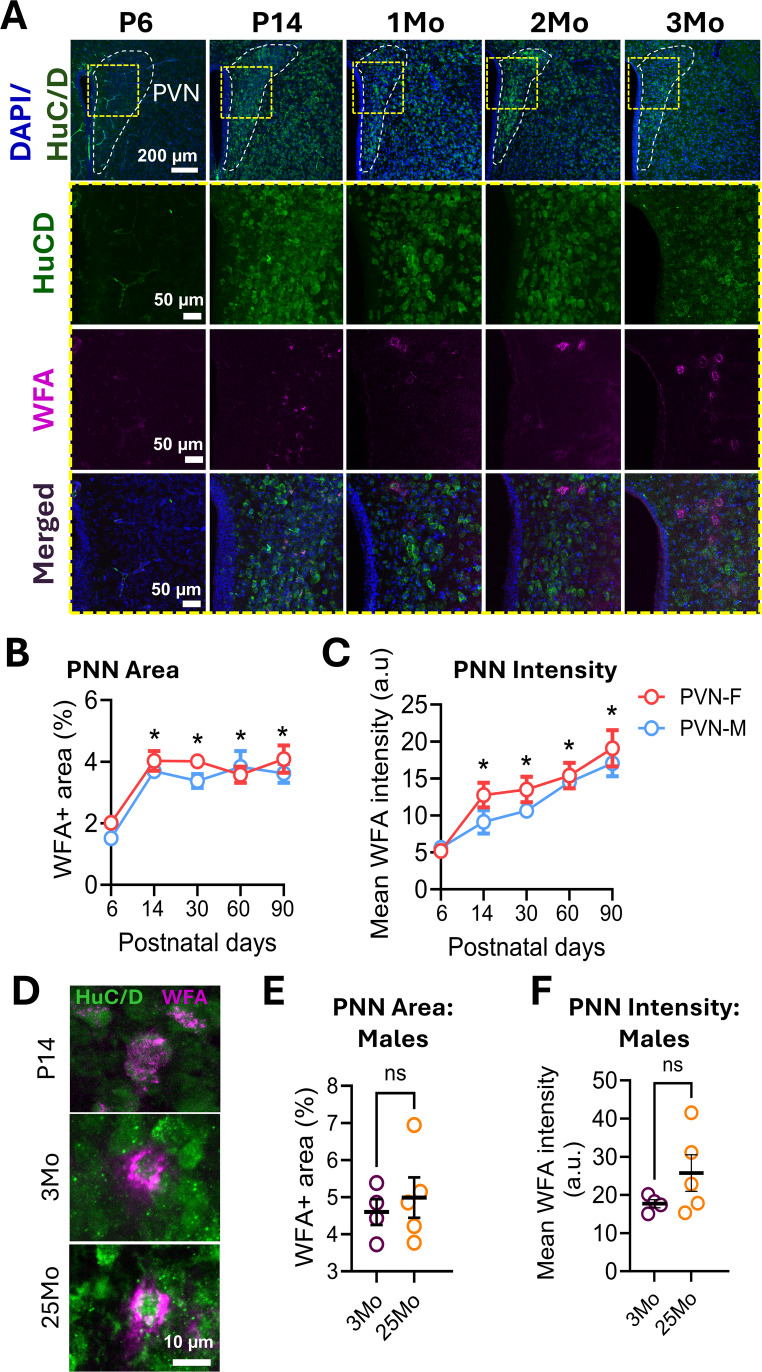
Developmental regulation of PNNs in the PVN. **a** WFA staining (magenta) shows no visible PNNs at P6, with progressive appearance at P14 and maturation by 1Mo. **b-c** Quantification of WFA+ area (Time, *p* < 0.0001; Sex, *p* = 0.1140; Interaction, *p* = 0.6546) and PNN intensity (Time, *p* < 0.0001; Sex, *p* = 0.0874; Interaction, *p* = 0.7332) across developmental timepoints (each data point = average of 3 mice, 7–12 sections per mouse). Two-way ANOVA with Tukey’s multiple comparisons. *p > 0.05 vs. P6 for each sex. PVN-F denotes females, and PVN-M denotes males. **d** Representative images of PNN-enwrapped neurons at P14, 3Mo, and 25Mo. **e-f** Quantification of WFA+ area (%) and fluorescence intensity in 3- vs. 25-month-old naïve male mice (3Mo *n* = 4; 25Mo *n* = 5). Data are shown as mean ± SEM; circles represent individual mice (average of 7–12 bilateral PVN sections/mouse). DAPI (blue) indicates nuclei and HuC/D (green) indicates neurons

PNNs follow a developmental trajectory across brain regions, typically emerging alongside circuit maturation and critical period closure (Carulli and Verhaagen 2021; Tewari et al. 2022). To assess this pattern in the PVN, we examined PNN across developmental time points using *Wisteria Floribunda Agglutinin* (WFA) staining, an established marker for PNNs (Härtig et al. 2022). WFA+ staining was absent at postnatal day 6 (P6) but appeared diffusely by P14 (Fig. 1a), suggesting that some PNN components are present but not organized as nets at this time. Quantification of WFA area (WFA+ area/total area; Fig. 1b) and total WFA intensity (including both condensed and diffuse labeling; Fig. 1c) revealed an increase in PNN components from P6 to all other time points in both sexes. No sex differences were detected at any time point.

PNNs form dense, net-like structures around somata and proximal dendrites (Balmer 2016; Cabungcal et al. 2013; Carceller et al. 2020; Carstens et al. 2016; Carulli and Verhaagen 2021; Celio 1993; Favuzzi et al. 2017; Fawcett et al. 2019; Frischknecht et al. 2009; Tewari et al. 2018). At P14, WFA+ labeling was mostly diffuse, in contrast to the compact PNNs seen at 1 month continuing to 3 and 25 months of age (Fig. 1a, d), indicating PNN maturation by 1 month of age. This developmental trajectory aligns with findings in other brain regions in mice (Gogolla et al. 2009; Mirzadeh et al. 2019; Pizzorusso et al. 2002) and in humans (Rogers et al. 2018). Interestingly, unlike cortical and hippocampal PNNs that often extend over dendrites, PVN PNNs showed limited dendritic coverage (Fig. 1d), similar to other hypothalamic areas (Alonge et al. 2020). Region-specific age-related changes in PNN density and intensity have also been reported (Brewton et al. 2016; Dong et al. 2023; Karetko-Sysa et al. 2014; Lehner et al. 2024; Yamada and Jinno 2013); however, we observed no significant differences in PNN area or intensity between 3 and 25 months male mice within the PVN (Fig. 1e, f).

## PNNs Surround Distinct Neuronal Subpopulations in the PVN

**Fig. 2:**
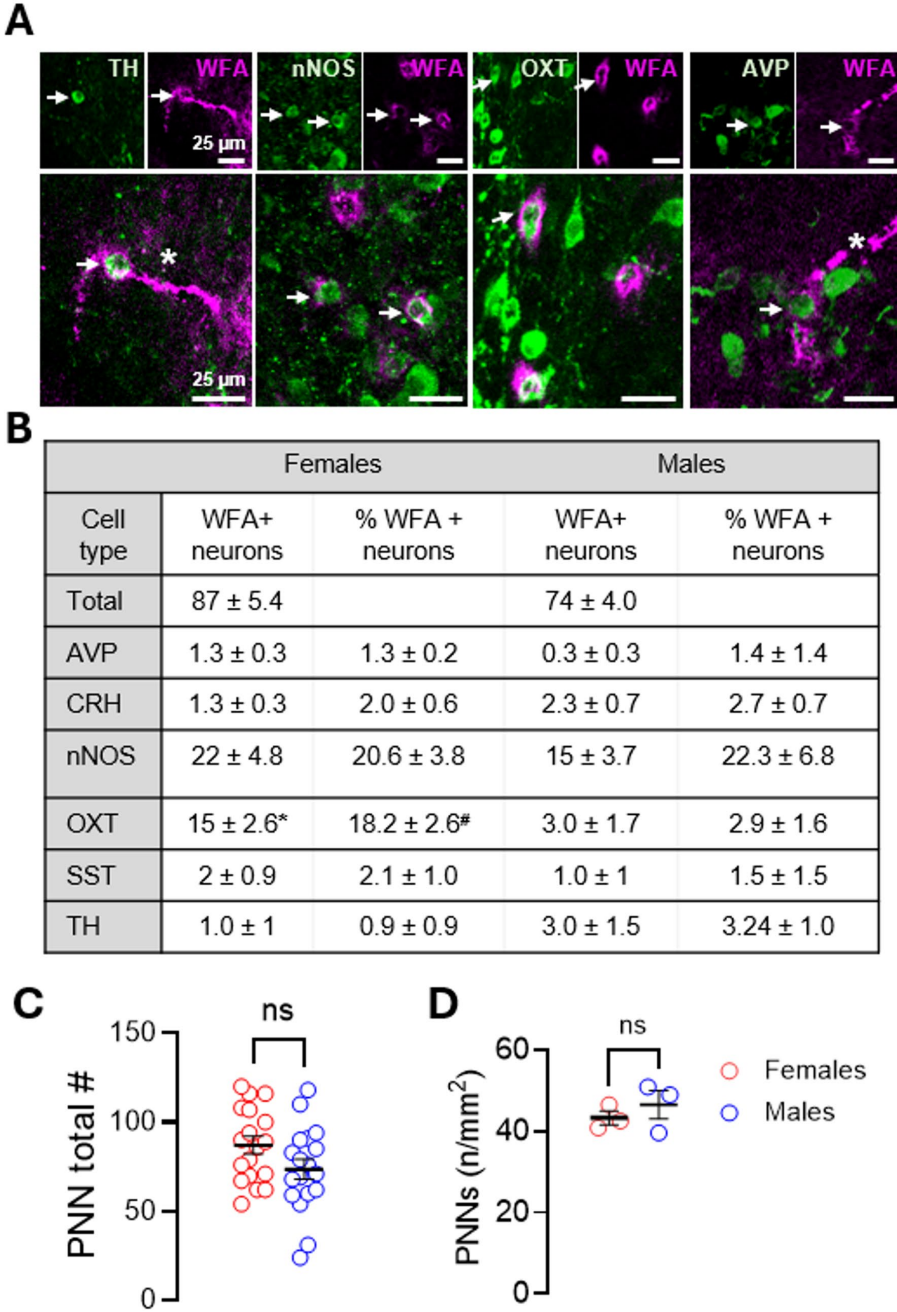
PNNs enwrap multiple neuronal cell types in the PVN. **a** Representative images of PNN-enwrapped neurons within the PVN. Arrows point to double-labeled neuron. Asterisk indicates WFA+ blood vessel. **b** Quantification of the number and percent of PNN-enwrapped neurons per cell type (*n* = 3/cell type). Parvalbumin (PV)-neurons were not detected within the PVN and are therefore not shown. **c** Distribution of absolute PNN number across all mice analyzed for neuronal cell type quantification in B (females vs. males: *n* = 18; 87.1 ± 4.9 vs. 73.6 ± 5.7, *p* = 0.0798). **d** PNN number normalized to PVN area in an independent cohort of 3-month-old mice (females vs. males: *n* = 3; 43.3 ± 1.7 vs. 46.5 ± 3.5, *p* = 0.4506, unpaired). Data are mean ± SEM; circles represent independent mice (average of 7–12 bilateral PVN sections/mouse). *p = 0.0169, ^#^*p* = 0.0077

Given the PVN’s complex neuronal makeup, we generated a stereological map to identify neuronal subtypes enwrapped by PNNs. Several subtypes exhibited PNN enwrapped neurons (Fig. 2a), with nNOS neurons being the most prevalent PNN+ subtype. Sex differences were only observed for oxytocin (OXT)-expressing neurons, with females exhibiting a higher number of total WFA+ neurons and %WFA+ neurons than males (Fig. 2b). The number of PNN-enwrapped neurons did not significantly differ between females and males either in absolute number (Fig. 2c) or when normalized to PVN area (Fig. 2d).

## DOCA-Salt Sensitive Hypertension Increases the Number of PNN-Enwrapped Neurons in the PVN of Male Mice

**Fig. 3:**
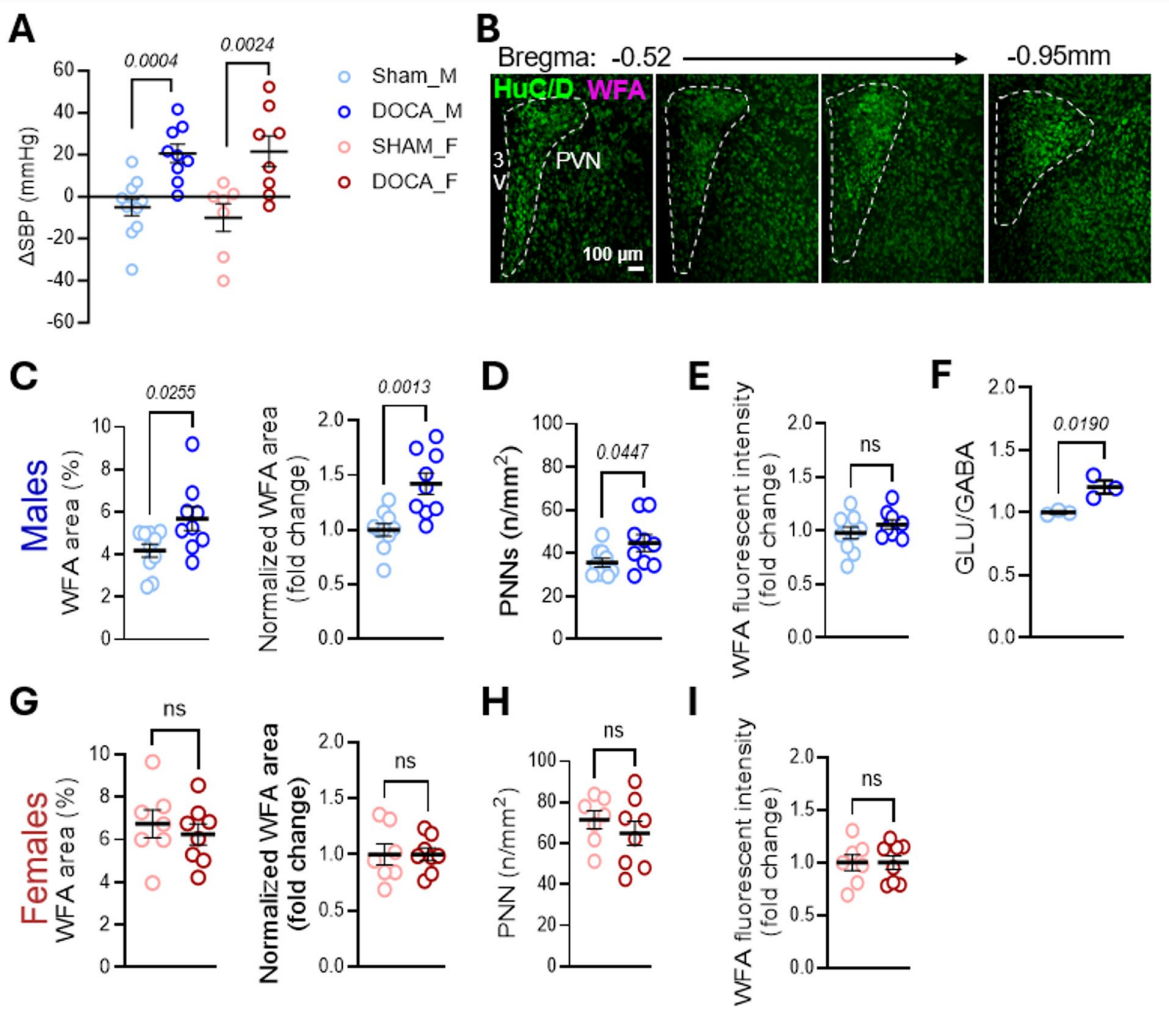
DOCA-salt increases PVN PNNs in male but not female mice. **a** Change in systolic blood pressure (SBP) after 21 days of DOCA-salt treatment (males: SHAM *n* = 11, DOCA *n* = 9, ∆SBP − 5.0 ± 4.1 vs. 20.6 ± 4.3, *p* = 0.0004, unpaired t-test; females: SHAM *n* = 12, DOCA *n* = 13, ∆SBP − 6.7 ± 4.7 vs. 21.0 ± 5.2, *p* = 0.0024, Mann-Whitney). **b** Schematic showing PVN sections included in quantification. **c-e** Quantification in males: **c** WFA area (%) and normalized WFA area (SHAM *n* = 10, DOCA *n* = 9; 4.2 ± 0.3 vs. 5.7 ± 0.5, *p* = 0.0255; normalized 1.0 ± 0.1 vs. 1.4 ± 0.1, *p* = 0.0013), **d** PNN number (35.6 ± 2.0 vs. 44.8 ± 3.9, *p* = 0.0447), and **e** WFA intensity (1.0 ± 0.1 vs. 1.1 ± 0.0, *p* = 0.2677). **f** Ratio of glutamate (Glu) to GABA (GABA) neurotransmitters after DOCA-salt. Pooled PVN tissue-punches from the PVN of both DOCA and SHAM male mice (*n* = 3; 2 mice/sample) (1.0 ± 0.0 vs. 1.2 ± 0.1, *p* = 0.0190). **g-i** Quantification in females: **g** WFA area (%) and normalized WFA area (SHAM *n* = 7, DOCA *n* = 8; 6.7 ± 0.7 vs. 6.3 ± 0.5, *p* = 0.5532; normalized 1.0 ± 0.1 vs. 1.0 ± 0.1, *p* > 0.9999), **h** PNN number (71.5 ± 4.4 vs. 64.9 ± 6.0, *p* = 0.4011), and **i** WFA intensity (1.0 ± 0.1 vs. 1.0 ± 0.1, *p* > 0.9999). Data are mean ± SEM; circles represent independent mice (average of 10–15 bilateral PVN sections/mouse)

We next examined the relationship between neurogenic hypertension and PNN expression in the PVN using the DOCA-salt model (Grobe et al. 2011). Both sexes exhibited a significant increase in BP after 21 days of DOCA-salt (Fig. 3a). Next, we quantified PNNs throughout the PVN (Fig. 3b). In males, DOCA-salt increased WFA + area in the PVN relative to sham, a result confirmed by cohort-normalized values (Fig. 3c). This increase corresponded to a higher number of PNNs (Fig. 3d). WFA intensity, however, was unchanged (Fig. 3e). This was associated with an increased ratio of glutamate to GABA in PVN tissue punches of DOCA-salt male mice (Fig. 3f), suggesting an increase in the E/I balance within this region. In contrast, female DOCA-salt mice showed no significant changes in WFA+ area, PNN count, or WFA intensity (Fig. 3g–i).

## Conclusions

We found that PNNs in the PVN follow the same developmental trajectory between males and female mice. The diffuse or weak WFA staining observed at P14 likely reflects secretion but not aggregation of core chondroitin sulfate proteoglycans (CSPGs) components and aligns with the development of PNNs in other brain regions (Lensjø et al. 2017; Yamada and Jinno 2013). In adults, we found that PNNs within the PVN have minimal dendritic coverage which is a feature also found in the arcuate nucleus (Alonge et al. 2020). Given the canonical role of PNNs in restricting synaptic plasticity when they are present in dendrites, we speculate that the lack of proximal dendrite coverage in the PVN may allow for greater plasticity aligning with the PVN’s role in integrating diverse brain inputs while maintaining somatic excitability. The most prevalent neuronal type enwrapped by PNNs was nNOS-expressing neurons in both males and females. We hypothesize that in the absence of PV+ neurons in both sexes, inhibitory neurons known to be preferentially enwrapped by PNNs, PNNs within the PVN preferentially enwrap the next larger inhibitory neuronal subtype (nNOS). Sex differences were observed only in OXT-enwrapped neurons. We speculate that this female-specific increase in OXT-enwrapped neurons may reflect sex differences in oxytocin circuit function rather than hormonal regulation of PNNs, as PNNs in the PVN are unaffected by gonadectomy (Zhang et al. 2021). However, future studies are needed to confirm these hypotheses.

Neurogenic hypertension was associated with a sex-specific increase in both the number and area of PNNs within the PVN of male mice. Despite an increase in BP in female mice, we did not observe changes in number, area, or intensity of PNNs. This suggests that PNNs may be involved in the increase in BP in male mice only. Of note, we did not assess the estrous cycle at the time of euthanasia in female mice. In some brain regions, but not all, PNNs are modulated by the estrous cycle (Nguyen et al. 2025). Thus, it is plausible that differences in PNNs would be observed if we controlled for estrous cycle at the time of euthanasia. An additional limitation of this study is its associative nature as we did not access BP regulation after manipulating PNNs within the PVN. This study establishes the first detailed characterization of PNNs in the PVN, and reports PNN remodeling in male DOCA-salt hypertension. Given the importance of PNN integrity for neuronal function, establishing this link provides a necessary foundation for the field to begin to consider this overlooked ECM component that regulates neuronal activity both in health and disease. Future research will leverage enzymatic and genetic techniques to determine whether causality exists between PNN integrity, neuronal activity within the PVN, and the regulation of BP.

In male mice exposed to the DOCA-salt, circumventricular organs provide increased excitatory input to the PVN, enhancing its activity (Guyenet 2006). Increased neuronal activity is associated with both the upregulation of PNN components and an increase in PNN-enwrapped neurons (Carulli et al. 2007; Dityatev et al. 2007). Thus, in DOCA-salt males, the early rise in neuronal activity may recruit previously “silent” neurons, enabling them to form PNNs. The emergence of these new PNNs could then stabilize and sustain their heightened activity, contributing to the persistent overactivation of the PVN observed in neurogenic hypertension. Supporting this concept, we confirmed an increase in the E/I ratio in the PVN of DOCA-salt treated male mice. Previous studies have also shown that neurogenic hypertension is associated with increased expression and secretion of corticotropin-releasing hormone (CRH) neurons (Goncharuk et al. 2002) as well as an increase in glutamatergic transmission (D.-P. Li et al. 2008; D.-P. Li and Pan 2006; Y.-F. Li et al. 2001; Ye et al. 2012). Future studies will focus on identifying which neuronal populations account for the increase in PNN-enwrapped neurons observed after DOCA-salt.

## Methods

### Animals

All procedures were approved by the Institutional Animal Use and Care Committee of Vanderbilt University Medical Center, protocol number M234000-00. C57BL/6J mice (Jax#664), and CRH-Cre mice (Jax#12704) crossed with Ai14-tdTomato reporter mice (Jax#7914) were used. Naïve C57BL/6J mice (P6–90 days, 3–5 months, and 25 months [NIA]) were used for developmental and DOCA-salt studies.

### DOCA-Salt Hypertension

Mice were acclimated to tail-cuff plethysmography (Hatteras MC4000) one week prior to surgery. Animals were randomized to sham or DOCA-salt groups; DOCA mice received a subcutaneous 50 mg DOCA pellet (Innovative Research of America, M-121), while shams underwent surgery without implantation. DOCA mice had free access to 0.9% NaCl in the drinking water; controls received water. Systolic blood pressure (SBP) was monitored twice weekly for 21 days. Change in SBP was calculated as final SBP (day 21) minus baseline (pre-surgery average).

### Developmental Map

P6 animals were anesthetized with 2% isoflurane and decapitated for brain tissue collection. All other animals were transcardially perfused with ice-cold PBS followed by 4% paraformaldehyde (PFA). All brains, including P6, were post-fixed in 4% PFA overnight, and cryoprotected in 30% sucrose in 1X PBS. Brains were sectioned at 40 μm using a vibratome (Leica VT1200S) and stored in 0.1 M phosphate buffer at 4 °C until used.

### Immunofluorescence

Free-floating sections were permeabilized (1X PBS/0.5% Triton X-100, 1 h), blocked (1X PBS/0.1% Triton X-100/10% NDS, 1 h), and incubated overnight with primary antibodies at 4 °C (1X PBS/0.1% Triton X-100/2% NDS): pre-conjugated WFA-FITC (1:300, Vector Laboratories FL-1351-2); HuC/D (1:500, ThermoFisher A-21271); Vasopressin (AVP, 1:500, Abcam AB213708); nNOS (1:300, Millipore Sigma AB5380); OXT (1:300, Abcam AB212193); Somatostatin (SST, ThermoFisher PA585759); Tyrosine hydroxylase (TH, ThermoFisher PA5-85167), Parvalbumin (PV, 1:300, Millipore Sigma MAB1572). The next day, sections were washed 3 times for 20 min each at room temperature. Sections were then incubated in secondary antibodies (PBS/0.1% Triton X-100/2% NDS) for 2 h at room temperature. Secondary antibodies against the primary host species were purchased from Jackson ImmunoResearch and were diluted 1:1000. Sections were mounted using VECTASHIELD Hardset Antifade Mounting Media with DAPI (Vector Laboratories, H-1500-10).

### Confocal Imaging Acquisition and Quantification

WFA-labeled sections were imaged with a laser scanning microscope (Zeiss LSM 880). Settings were kept constant across all groups (20X, 2 μm z-stacks at room temperature; pixel size: 2.37 μm; zoom: 0.7; pinhole: 90; digital gain: 1). Bilateral images of the PVN (bregma − 0.52 to −0.95; 7–15 per mouse) were analyzed using ImageJ. PNN-positive neurons were identified and counted. PNN intensity was quantified using the manual method by *Slaker et al.* (Slaker et al. 2016), with SUM-slice projections, background subtraction, and thresholding.

### Liquid Chromatography/Mass Spectrometry Analysis of Neurotransmitters

Mice were anesthetized in 2% isoflurane, euthanized, and brain tissue collected and sectioned into 1 mm sections using a metal brain mold. Tissue punches (0.75 mm diameter) containing the PVN from 2 mice were flash-frozen, pooled, and kept on −80 °C until analysis. Neurotransmitters glutamate and GABA were measured by the Vanderbilt Neurochemistry Core (VNC). Briefly, tissue punches were homogenized, 10 analytes of interest were quantified simultaneously from each sample using LC/MS, and analyte concentrations were normalized to total protein content of the sample as previously described (Brown et al. 2023).

### Statistical Analysis

Data were analyzed in GraphPad Prism 10. Normality was assessed (Shapiro-Wilk), outliers were removed (Grubbs test), followed by unpaired two-tailed t-tests. Two-way ANOVA followed by Tukey’s multiple comparison was performed for Fig. 1b and c. *p* < 0.05 was considered significant.

## Data Availability

Data is provided within the manuscript. Raw or analyzed data during the current study will be available from the corresponding authors on reasonable request.
